# Bright’s Disease and Allied Affections of the Kidneys

**Published:** 1886-09

**Authors:** 


BRIGHT'S DISEASE AND ALLIED AFFECTIONS OF THE KIDNEYS. By Charles W. Purdy, M. D., Queen's University, Professor of Genito-Urinary and Renal Diseases in the Chicago Polyclinic, etc. 8vo., 288 pages, with 18 illustrations. Cloth, $2. Philadelphia: Lea Brothers & Co. 1886.

This book is a systematic, practical and concise description of the pathology and treatment of the chief organic diseases of the kidneys associated with albuminuria.

The author has given, and we think justly so, more prominence to scarlatinal and puerperal nephritis than has heretofore been done by writers on this subject, and it will be of great value to the physician who is called on to treat these cases.

It is the most practical work on the subject of albuminuria we know, and should be in the hands of every practitioner in this country.

